# Perspectives on Medical Education Special Edition: Lessons learned from health professions education scholarship failures surprises

**DOI:** 10.1007/s40037-017-0355-z

**Published:** 2017-05-31

**Authors:** Lara Varpio, Alisa Nagler

**Affiliations:** 1 Uniformed Services, University Bethesda, Bethesda, MD USA; 2 American College of Surgeons, Chicago, IL USA; 3 Duke University School of Medicine, Durham, NC USA

## A call for surprises

We’ve all tried things that didn’t work. We’ve enthusiastically introduced a flipped classroom format that the learners didn’t flip over. We’ve carefully crafted research projects only to realize that the hypothesis was woefully ill-conceived. We’ve implemented new assessment strategies that generated lots of data, but left us with little insight into student performance. We’ve all been there.

However, it is often through these moments of failure that we gain real in-depth understanding of a phenomenon, that we realign our perspectives in important ways, and that we experience our most resonant ‘a-ha’ insights. Indeed, sometimes our mistakes are more informative than our successes.

Problematically, there are few opportunities to share these lessons learned with the broader community. Where in the literature are we able to report a failure that revealed a surprising lesson or novel insight? It is precisely this gap that we hope to fill with a special edition of *Perspectives on Medical Education. *We are inviting health professions education scholars to share the stories of what they learned from these surprises, when things did not turn out as expected.

We invite you to write about any health professions education experience (be it in educational practice or research), from across the educational continuum (i. e., undergraduate, graduate, and continuing education), that didn’t go as planned, but left you with new insights and/or perspectives. We are soliciting papers that share the intention of the original effort (i. e., we tried ‘a’ because ‘b’), what went wrong (i. e., it didn’t work because of reason ‘c’), and what was learned from the experience (i. e., the lesson learned was ‘d’ that helped us move forward in ‘e’ ways). These are *not* papers for complaints or lamentations. These are papers about the insights gained from surprising outcomes, mistakes or failures, and the lessons the community can take away from your experience.

We appreciate that sharing the stories of failures and surprises may be uncomfortable. Fortunately, since failure is a universal experience, some of our community’s most acclaimed scholars from around the world have already submitted their manuscripts to populate this special edition. In the company of scholars like Jen Cleland, Tim Wilkinson, Fedde Scheele and Geoff Norman, we hope that you too will be comfortable sharing the lessons you learned when things didn’t go according to plan. You can find two examples of the manuscript format illustrating the kinds of stories we hope you will share here (DOI: 10.1007/s40037-017-0354-0, DOI: 10.1007/s40037-017-0356-y).

### Manuscript format

(Manuscripts should not exceed 2500 words. Please use these headings in your manuscript)**:**

- **The story:** Describe what you tried and why you tried it.
- **Surprising outcomes: **Describe the unexpected or unplanned consequences of that effort, or the ‘negative’ results. Clarify what went wrong.
- **Lessons learned: **Explain why the surprise, mistake or failure occurred. Describe the insights you gained and why they are important and relevant to others.
- **Moral of the story: **Share what you learned from this experience that may be helpful to others. (We recognize it may have little to do with your initial rational for the effort and/or research question).

## Timeline

Papers are due no later than: 1 October 2017

Review decisions are announced: 1 December 2017

Revisions are made and re-submitted by: 1 February 2018

Final acceptance decisions are announced: 8 February 2018

Publication date: June 2018

